# The Formation of pH-Sensitive Wormlike Micelles in Ionic Liquids Driven by the Binding Ability of Anthranilic Acid

**DOI:** 10.3390/ijms161226096

**Published:** 2015-11-26

**Authors:** Qing You, Yan Zhang, Huan Wang, Hongfu Fan, Jianping Guo, Ming Li

**Affiliations:** 1 School of Energy Resources, China University of Geosciences, Beijing 100083, China; agewanghuan@163.com (H.W.); fanhongfu@126.com (H.F.); guojp@cugb.edu.cn (J.G.); 2 Sinopec Shengli Oilfield Research Institute of Petroleum Engineering, Dongying 257000, China; liming521.slyt@sinopec.com

**Keywords:** pH-sensitive, wormlike micelles, rheology, morphological variation, formation mechanism

## Abstract

Wormlike micelles are typically formed by mixing cationic and anionic surfactants because of attractive interactions in oppositely charged head-groups. The structural transitions of wormlike micelles triggered by pH in ionic liquids composed of *N*-alkyl-*N*-methylpyrrolidinium bromide-based ILs (ionic liquids) and anthranilic acid were investigated. These structures were found responsible for the variations in flow properties identified by rheology and dynamic light scattering, and account for the structures observed with cryogenic transmission electron microscopy (Cryo-TEM). High-viscosity, shear-thinning behavior, and Maxwell-type dynamic rheology shown by the system at certain pH values suggested that spherical micelles grow into entangled wormlike micelles. Light scattering profiles also supported the notion of pH-sensitive microstructural transitions in the solution. Cryo-TEM images confirmed the presence of spherical micelles in the low-viscosity sample and entangled wormlike micelles in the peak viscosity sample. Nuclear magnetic resonance spectroscopy analysis revealed that the pH sensitivity of ionic liquid systems originated from the pH-dependent binding ability of anthranilic acid to the cationic headgroup of ionic liquids.

## 1. Introduction

Molecular self-assembly is a kind of entity with a special structure, which associates spontaneously by molecules [1,2,3,4,5,6]. It not only promotes the development of functional and complex materials, but also leads to the formation of different aggregate morphologies such as crystalline structures, wormlike micelles, vesicles, and spherical micelles [7,8,9]. Among them, wormlike micelles (WLMs) have drawn much attention from researchers all over the world because of their outstanding performance and extensive applications [10,11,12,13]. When the concentration of surfactant exceeds a threshold concentration (C*), surfactant molecules may assemble and form dynamic polymer structures and the solution then exhibits greatly altered macroscopic viscoelastic properties. However, the essential difference between the polymers with covalent bonds and WLMs is that the bonds are transient in the latter case. The self-regenerating nature of WLMs after subjecting these systems to flow or deformation makes them extremely useful in many industrial applications including oil production, drag reduction agent, and drug delivery [14,15,16].

Recently, smart wormlike micelles (SWLMs) have attracted considerable interest due to the tunability of their viscoelasticity with imposed stimuli, such as electric currents, UV-vis, temperature, redox reaction, and pH [17]. Among these perturbations, pH variation has attracted particular interest since this pH control is relatively easy to introduce and control. Thus far, the synthesis of peculiar molecules with functional groups and the introduction of a pH-sensitive monomer molecular into the system are the most widely used methods to prepare the pH-sensitive materials. Ionic liquids (ILs) are also known as a kind of low temperature molten salt, whose melting point is lower than 100 °C [18]. Additionally, the advantages of ILs also include high thermal stability, high solubility, negligible vapor pressure, wide electrochemical windows, *etc*. Among them, the most fascinating feature of ILs is the highly modified characteristics with being simply adjusted to the chemical structures of the head-group. Recently, many studies have reported that some ILs can perform molecular self-assembly in aqueous solutions as a result of ILs possession of natural amphiphilic properties. Nevertheless, the pH-sensitive self-assembling structures are rarely reported when formed by ILs. Therefore, we intend to test the feasibility of creating pH-sensitive controlled ILs by utilizing the highly modified characteristics of ILs.

In the present study, we have designed pH-sensitive ILs composed of *N*-alkyl-*N*-methylpyrrolidinium bromide-based ILs and anthranilic acid. Multiple techniques including dynamic light scattering, rheological measurements, nuclear magnetic resonance spectroscopy, and cryogenic-transmission electron microscopy were employed to examine the properties of self-assembly. In addition, the mechanism of self-assembling structural changes and the variety of pH values has been discussed.

## 2. Results and Discussion

### 2.1. Rheological Properties of C_16_MPBr-AA Complex System

AA is hardly soluble in water due to its strong hydrophobicity. But its solubility would be enhanced with the presence of C_16_MPBr micelles. When *C*_C16MPBr_/*C*_AA_ = 0.1:1, the quantity of AA is too excessive to be completely dissolved. When *C*_C16MPBr_/*C*_AA_ goes up to 1:1, the AA solid would completely disappear from the bottom of the test tube. Therefore, the molar ratio of C_16_MPBr/AA for the following experiments was set as 1:1 to ensure homogeneity of the solutions for further measurements.

**Figure 1:**
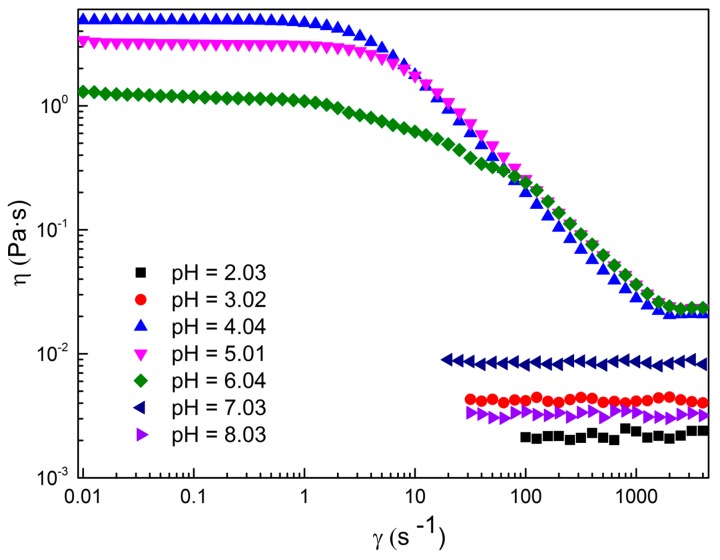
Steady shear viscosity *versus* shear rate γ (with a dot over the symbol) plots for 80 mM C_16_MPBr-AA aqueous solutions with different pH values.

**Figure 2:**
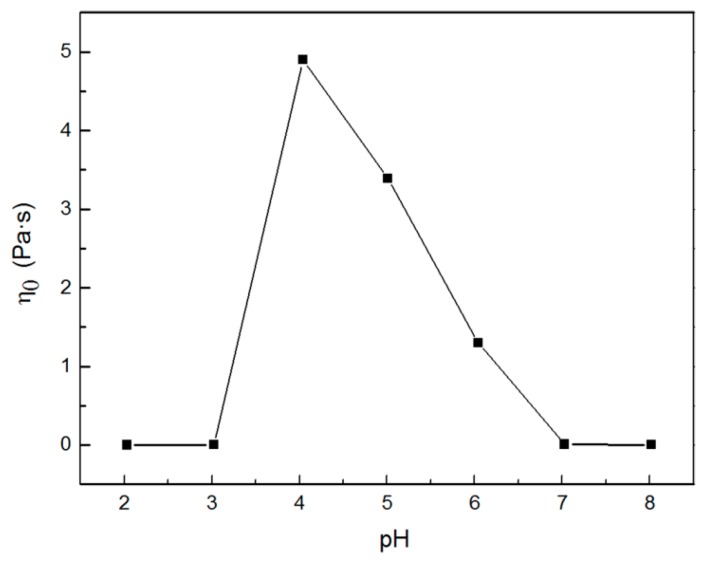
Variations of zero-shear viscosity (η_0_) as a function of 80 mM C_16_MPBr-AA aqueous solution with different pH values.

Figure 1 exhibits the shear viscosity variation of the 80 mM C_16_MPBr/80 mM AA system as a function of the shear rate with different pH values. As can be seen, the shear viscosity of the sample at pH 2.03 keeps a small constant no matter how the shear rate changes. This particular phenomenon is usually considered a typical characteristic of Newtonian fluids. One can also observe a similar phenomenon with a slight increase of viscosity for the sample at pH 3.02. By contrast, the shear viscosity curve of the sample at pH 4.04 shows the feature of Newtonian fluids in the low shear rate region and a shear thinning phenomenon in the high shear rate region simultaneously, which is usually regarded as the formation of WLMs with a structural alignment at the high shear rate [19]. What is noteworthy is that when the pH of the sample reaches 5.01, the sample cannot flow freely when it inverses the tube for a long time, showing the properties of “gel-like” behavior similar to the one which is usually found in polymer solutions. As the pH exceeds 5.01, the viscosity drops significantly. The zero-shear viscosity (η_0_) has been obtained from the shear rate dependence of the steady shear viscosity. As a function of the pH values, η_0_ is depicted in Figure 2. It is obvious that η_0_ values increase with the increasing of pH firstly and exists at a maximum η_0_ value at pH = 4.04, followed by a significant decrease.

**Figure 3:**
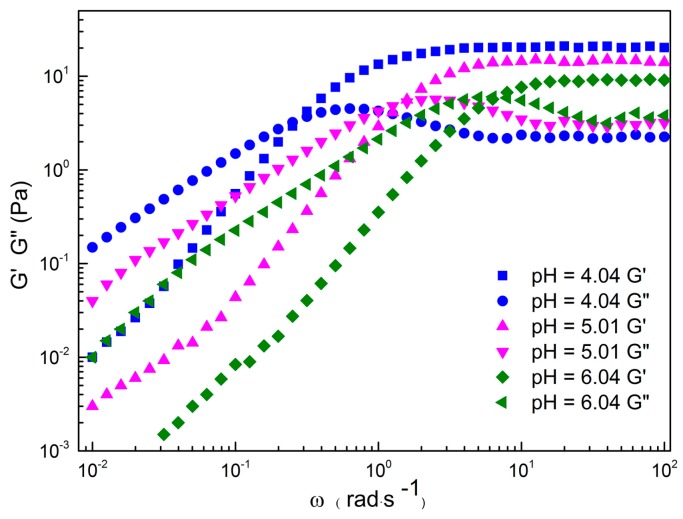
The relationship of *G′* and *G″ versus* oscillation frequency ω for 80 mM C_16_MPBr-AA aqueous solution with different pH values.

The storage modulus (*G′*) and loss modulus (*G″*) of the 80 mM C_16_MPBr/80 mM AA system as a function of oscillatory shear frequency (ω) at different pH values are plotted in Figure 3. Within the pH range of 4.04–6.04, *G′* crosses and prevails over *G″* when it exceeds a critical shear frequency (ω_c_). In other words, the typical viscous behavior of the sample would emerge at a low frequency zone; while an evident elastic response would appear at high frequency zone.

The dynamic rheological behaviors of WLMs are usually described with the Maxwell model on the basis of the following equations [20]:

(1) $$G^{\prime }=\frac{{\left(\omega \tau_{R}\right)}^{2}}{1+{\left(\omega \tau_{R}\right)}^{2}}G_{0}$$

(2) $$G^{\prime \prime }=\frac{\omega \tau_{R}}{1+{\left(\omega \tau_{R}\right)}^{2}}G_{0}$$

where, when the 80 mM C_16_MPBr/80 mM AA system at pH 4.04~6.04, the data of *G′* and *G″* conform to Maxwell equation in the low shear frequency region. The typical viscoelastic fluid behaviors of WLMs indicate that the persistence time became long, acting more like conventional polymer solutions where the bonds mimic essentially irreversible chemical reactions, which verifies again the presence of WLMs [21]. While in the high shear frequency region, the deviation of *G′* and *G″* from the Maxwell model can usually be identified as a transition in relaxation mode with a distinct time scale.

**Figure 4:**
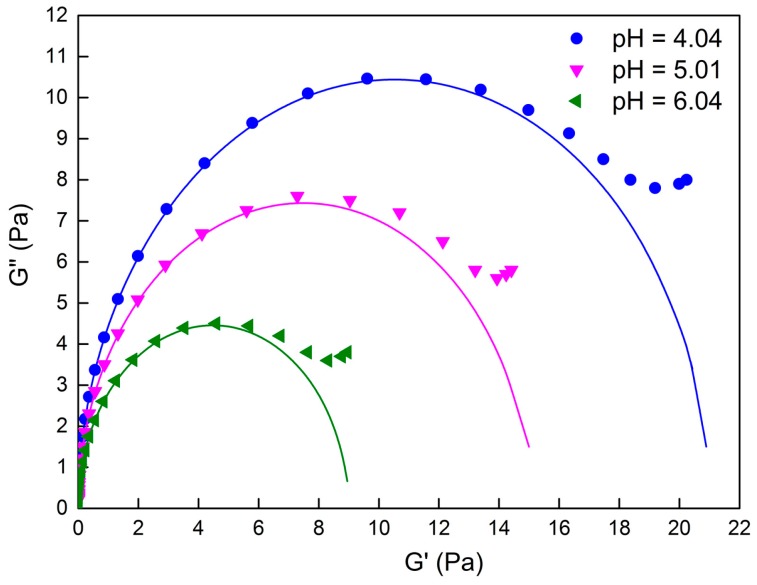
The variation of *G″* as a function of *G*′ of 80 mM C_16_MPBr-AA aqueous solution with different pH values.

The Cole-Cole plot (plot of G″ as a function of G′) is generally considered as the criteria to determine whether it follows the Maxwell model. As shown in Figure 4, the points are the actual experimental data, while the solid lines represent theoretical values, which are calculated. The data was plotted as semicircles at low frequency, but then deviate at high frequency, which indicates the formation of worm-like micelles [22]. This phenomenon is generally interpreted as the existence of Rouse modes or “breathe modes” [23], which is usually observed in other viscoelastic solutions [24].

### 2.2. Morphological Variation of Self-Assembly Induced by pH

**Figure 5:**
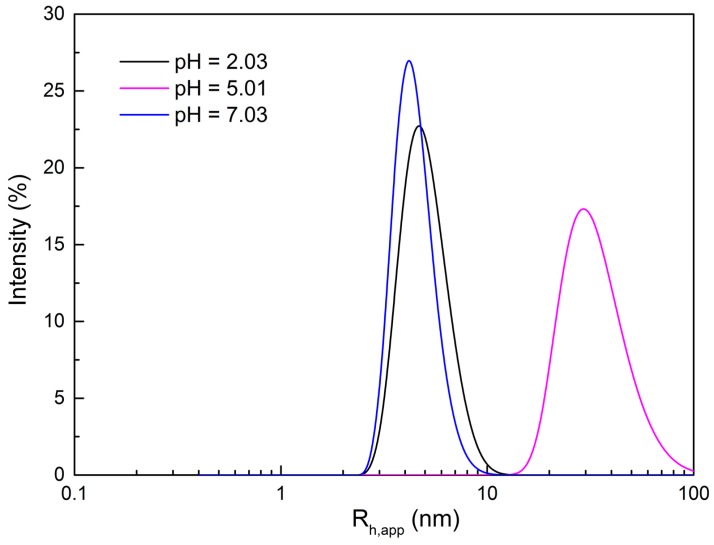
Size distribution of 80 mM C_16_MPBr-AA aqueous solution with different pH values.

As discussed above, the pH-sensitive rheological behavior of the 80 mM C_16_MPBr/80 mM AA system is attributed to the transformation from a spherical micelle morphology to a Worm-like one. To corroborate this, DLS methods were characterized for 80 mM C_16_MPBr/80 mM AA solutions with different pH values. Figure 5 reveals the hydrodynamic diameter and size distribution of the aggregates in solutions with various pH values. It can be clearly seen that the average hydrodynamic diameter of the micelles in the system increased obviously with the increase of pH, and was followed by a decrease. The results of DLS are in accord with the shear viscosity variation. Although the real size of the micelles cannot be calculated according to the result of the apparent hydrodynamic diameter due to strong electrostatic interactions among micelles, the general tendency of the micellar morphology can also be confirmed [25].

**Figure 6:**
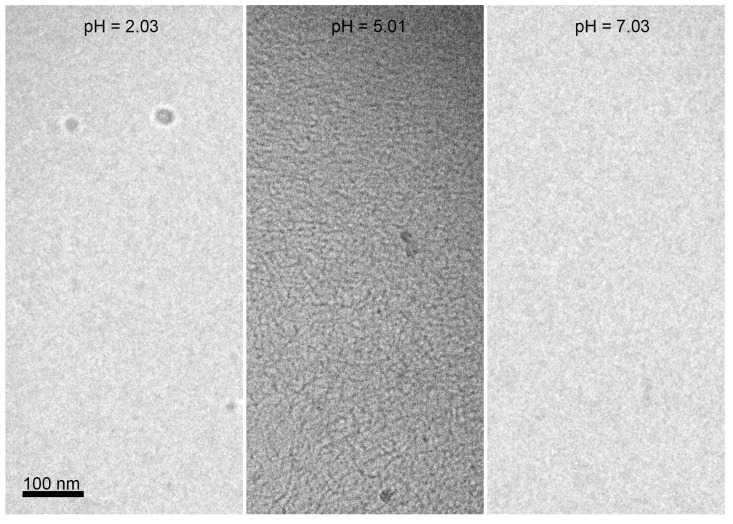
Cryo-TEM micrographs of nanostructured fluid based on 80 mM C_16_MPBr-AA aqueous solution at different pH conditions.

In addition, cryo-TEM was employed to visualize the micelle morphology of 80 mM C_16_MPBr/80 mM AA solutions with different pH values. From Figure 6, elongated WLMs were evidenced at pH 5.01 and disappeared at pH 2.03 and pH 7.03. Thus, the remarkable rheological changes of 80 mM C_16_MPBr/80 mM AA solutions are supposed to be the morphological transition from spherical micelles (pH = 2.03) to WLMs (pH = 5.01) and then to spherical micelles (pH = 7.03).

### 2.3. Possible Mechanism of the Morphological Variation Induced by pH

**Figure 7:**
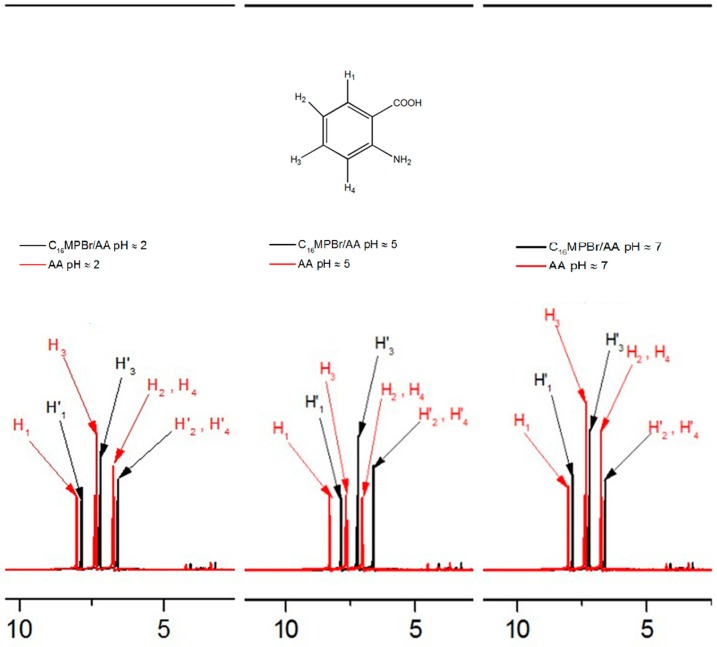
The ^1^H-NMR spectrum of 80 mM AA in the presence and absence of 80 mM C_16_MPBr at different pH values.

**Figure 8:**
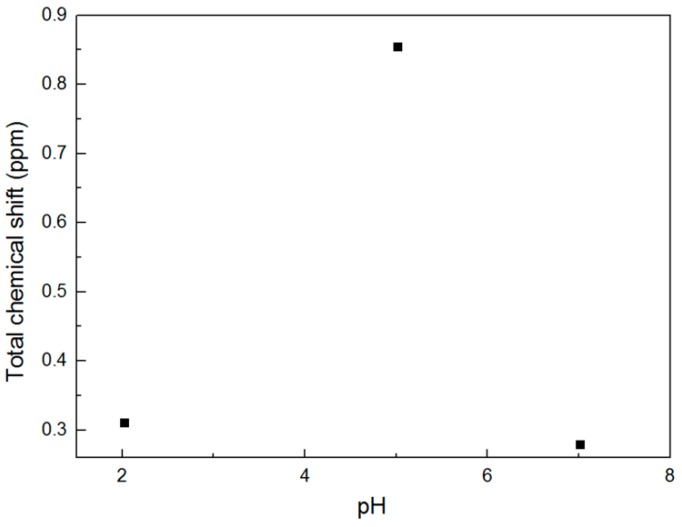
Total chemical shifts of characteristic ^1^H-NMR signals measured at various pH values.

It is known that AA contains a carboxylic group, which is pH responsive in the aqueous solution. The p*K*_a_ values of the AA are 2.14 and 4.92 and its isoelectric point is 3.2. The molecule structure of AA in the aqueous solution can be precisely controlled by changing the pH on either side of IEP. Based on this, the interactions between C_16_MPBr and AA are significantly affected and subsequently influence the aggregate structure of ILs. To further investigate the influence on the aggregate structure of ILs, ^1^H-NMR measurements were carried out in the conditions of different pH values. Typical ^1^H-NMR spectra of C_16_MPBr/AA system at three pH values are shown in Figure 7. Figure 8 depicts that the chemical shift of H1 and H2 protons with the increase of the pH value. It firstly shifts to downfield when pH below ≈5 and then shifts upfield. The ^1^H-NMR chemical shifts indicate the variation in the distance between the polar head groups of surfactant molecules and the variation of the micellar morphology [26]. In general, a downfield shift represents that the electron density of the environment declines gradually [27]. This can lead the protons to the headgroups of ILs. When pH value increases from ≈2 to ≈5, the gradual decline of the electron density suggests an increased binding of AA on the headgroups of C_16_MPBr. The electrostatic repulsion between C_16_MPBr and AA molecules was largely screened, which induced the morphology of aggregates transit from spherical micelles to WLMs. Conversely, an upfield shift manifested that the electron density of the environment goes up gradually [28,29]. Therefore, the electrostatic repulsion between C_16_MPBr and AA molecules is incapable of shielding effectively when the pH value further increases , and a reasonable explanation about the morphology of aggregates transit in reverse from WLMs to spherical micelles has been given. Therefore, it seems likely that the pH-sensitive SWLMs are composed of *N*-alkyl-*N*-methylpyrrolidinium bromide-based ILs, and anthranilic acids originated from the pH-dependent binding ability of AA to the cationic headgroup of ILs.

### 2.4. pH-Sensitive Ability of C_16_MPBr-AA Complex System

**Figure 9:**
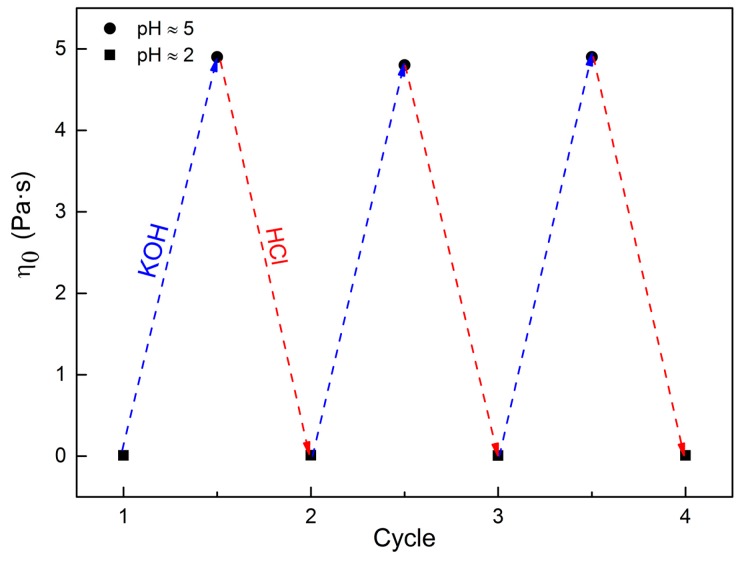
Switchable viscosity of 80 mM C_16_MPBr-AA aqueous solution at different pH values.

In real applications, the pH-sensitivity of viscoelasticity is supposed to repeat several times. Two pH values (≈2 and 5) were chosen to verify the pH-sensitive ability due to their low and high viscosity. As shown in Figure 9, when the pH value of the sample is equal to 2, the viscosity is approximately equal to 5 mPa·s; while when the pH value of the sample is increased to 5, the viscosity rises significantly up to 4900 mPa·s, which is about 10^3^ times that of the viscosity when the pH value is 2. In addition, the high viscosity would immediately get back to the initial viscosity when the pH value is adjusted to 2, and such a reversible process can be easily achieved for more than three times without any deterioration. This reversible switch ability is remarkable and has significant practical value in many industrial fields.

## 3. Materials and Methods

### 3.1. Materials

**Figure 10:**
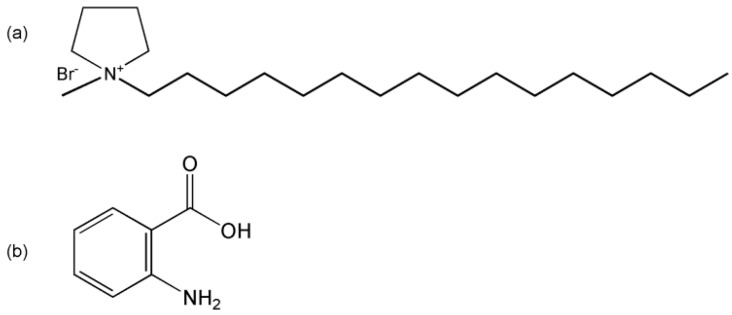
The molecular structure of *N*-hexadecyl-*N*-methylpyrrolidinium bromide (C16MPBr) (**a**) and anthranilic acid (AA) (**b**).

The IL *N*-hexadecyl-*N*-methylpyrrolidinium bromide (C_16_MPBr) was synthesized and purified as described previously [30]. The C_16_MPBr was dried in vacuum conditions at 25 °C for one week. Anthranilic acid (AA) was purchased from the Aladdin Chemistry Company (Shanghai, China). The molecular structures of C16MPBr and AA are shown in Figure 10. Sodium hydroxide and hydrochloric acid are analytical reagent grade products of Sinopharm Chemical Reagent Company (Shanghai, China). These chemicals were used as received. Hydrochloric acid or sodium hydroxide aqueous solutions were used to adjust the required pH of samples. The pH of the samples was determined by a Sartorius PB-10 pH meter. All the samples were equilibrated for 48 h before each measurement.

### 3.2. Rheological Measurements

Rheological measurements were performed on a HAAKE MARS III rotational rheometer equipped with a CC26Ti cylindrical rotor at 25 °C. For steady shear experiments, the shearing rate was set to a range of 0.01–1000 s^−1^. In the oscillatory mode, the frequency region was 0.01 to 100 rad∙s^·1^. The linear viscoelastic region was set as 1.0 Hz according to a dynamic strain sweep test.

### 3.3. Nuclear Magnetic Resonance (NMR) Spectroscopy

A Bruker AVANCE III 600 MHz spectrometer (Rheinstetten, Germany) was used to record ^1^H-NMR spectra with the solvent of D_2_O at 25 °C. The water signal was suppressed by presaturation method.

### 3.4. Dynamic Light Scattering (DLS) Measurements

DLS analyses were carried out on a Malvern Zetasizer Nano ZS instrument (Malvern, UK) equipped with a solid-state He−Ne laser. The wavelength of the incident beam was set at 632.8 nm and the scattering angle was set at 90 °C. To ensure temperature homogeneity of the samples, each sample was required to equilibrate at 25 °C for 15 min. Each sample needed to be tested for three times to ensure the reproducibility of DLS results.

### 3.5. Cryogenic-Transmission Electron Microscopy (Cryo-TEM)

Cryo-TEM images of samples were prepared with a 120KV JEM-1400 Plus TEM instrument (Tokyo, Japan) with a Gatan US1000 894 CCD monitor. The cryo-TEM samples were stored in a controlled environment vitrification system (Tokyo, Japan). Firstly, approximately 5 mL of the sample was added onto a perforated polymer film. After 10 s, the polymer film was immediately immersed into a −165 °C liquid ethane reservoir. The sample would be kept in liquid nitrogen environment until observation.

## 4. Conclusions

In summary, we have reported that the fabrication of pH-sensitive SWLMs is composed of *N*-alkyl-*N*-methylpyrrolidinium bromide-based ILs and anthranilic acid. The morphology of wormlike micelle transforms between spherical micelles and WLMs, which was revealed by the rheology, cryo-TEM, and DLS methods. With the aid of ^1^H-NMR spectroscopy analysis, one can conclude that the pH-sensitive SWLMs are composed of *N*-alkyl-*N*-methylpyrrolidinium bromide-based ILs and anthranilic acid, and this is ascribed to the pH-dependent binding ability of AA to the cationic headgroup of ILs. This work not only helps deepen the understanding of the formation mechanism of SWLMs, but it also broadens the application of pH-sensitive materials in the fields of oil and gas, enhanced oil recovery material systems, *etc*.
